# Analysis of the Human Prostate-Specific Proteome Defined by Transcriptomics and Antibody-Based Profiling Identifies TMEM79 and ACOXL as Two Putative, Diagnostic Markers in Prostate Cancer

**DOI:** 10.1371/journal.pone.0133449

**Published:** 2015-08-03

**Authors:** Gillian O'Hurley, Christer Busch, Linn Fagerberg, Björn M. Hallström, Charlotte Stadler, Anna Tolf, Emma Lundberg, Jochen M. Schwenk, Karin Jirström, Anders Bjartell, William M. Gallagher, Mathias Uhlén, Fredrik Pontén

**Affiliations:** 1 Department of Immunology, Genetics and Pathology, Science for Life Laboratory, Uppsala University, Uppsala, Sweden; 2 UCD School of Biomolecular and Biomedical Science, UCD Conway Institute, University College Dublin, Belfield, Dublin 4, Ireland; 3 OncoMark Ltd, NovaUCD, Belfield Innovation Park, Belfield, Dublin 4, Ireland; 4 Science for Life Laboratory, KTH—Royal Institute of Technology, Stockholm, Sweden; 5 Department of Clinical Sciences Lund, Oncology and Pathology, Lund University, Skåne University Hospital, Lund, Sweden; 6 Department of Clinical Sciences, Division of Urological Cancers, Skåne University Hospital Malmö, Lund University, Malmö, Sweden; University of Kentucky College of Medicine, UNITED STATES

## Abstract

To better understand prostate function and disease, it is important to define and explore the molecular constituents that signify the prostate gland. The aim of this study was to define the prostate specific transcriptome and proteome, in comparison to 26 other human tissues. Deep sequencing of mRNA (RNA-seq) and immunohistochemistry-based protein profiling were combined to identify prostate specific gene expression patterns and to explore tissue biomarkers for potential clinical use in prostate cancer diagnostics. We identified 203 genes with elevated expression in the prostate, 22 of which showed more than five-fold higher expression levels compared to all other tissue types. In addition to previously well-known proteins we identified two poorly characterized proteins, TMEM79 and ACOXL, with potential to differentiate between benign and cancerous prostatic glands in tissue biopsies. In conclusion, we have applied a genome-wide analysis to identify the prostate specific proteome using transcriptomics and antibody-based protein profiling to identify genes with elevated expression in the prostate. Our data provides a starting point for further functional studies to explore the molecular repertoire of normal and diseased prostate including potential prostate cancer markers such as TMEM79 and ACOXL.

## Introduction

Prostate specific antigen (PSA) has emerged as a useful tumor marker in oncology and PSA-based screening is widely used despite a relative lack of both specificity, leading to overdiagnosis and treatment of early stage prostate cancer, and sensitivity, leading to prostate cancer not being detected early enough [1–5]. Thus there is a need for better markers for early detection of prostate cancer.

PSA is a serine protease and one of three most abundant proteins secreted from the prostate gland [6]. In the malignant prostate, tissue architecture is abnormal which facilitates PSA leakage to capillaries in the stromal compartment. Non-malignant prostate conditions, including prostatitis and benign prostatic hyperplasia (BPH), can lead to elevated serum PSA, limiting the specificity of PSA elevation for cancer detection [7]. Thus, determining which patients require further examination with transrectal ultrasonography (TRUS)-guided biopsies remains a significant problem.

Several other markers have been implicated as potential biomarkers of prostate cancer, such as alpha-methylacyl coenzyme A racemase (AMACR) which has been shown to be significantly up-regulated in prostate cancer and detectable in both serum and cancer tissue. Other such diagnostic biomarkers include prostate carcinoma mucin-like antigen (PMA), GOLM1, fatty acid synthase (FASN), TMPRSS2-ERG fusion prostate cancer antigen 3 (PCA3), KLK3, KLK2, HOXB13, GRHL2 and FOXA1[8–12]. However, up-to-date, no individual marker has proven better than PSA.

Prostate cancer is diagnosed based on histopathological examination of multiple TRUS-guided prostatic core biopsies. The identification of cancer in the prostate is prone to subjectivity and error due to the reliance on human interpretation and that biopsies only provide a small amount of tissue, which often includes only a few malignant glands and histological benign mimics of cancer. The discovery of a specific marker of either prostate cancer or benign prostatic glands that also could be measured in serum would be beneficial to avoid unnecessary invasive diagnostic tests.

The interpretation of quantitative transcriptomics data based on mRNA sequencing of tissue samples is a challenge due to the heterogeneity of cell types that comprise various tissue types. Here we have analyzed genes expressed in normal human prostate and compared these data to the trancriptomes of 26 other normal human tissue types based on recently published RNA-seq data [13]. The transcriptomics analysis was combined with immunohistochemistry-based protein profiling data available from the Human Protein Atlas (www.proteinatlas.org) [14, 15] to provide a map of gene expression on both the RNA and protein level in the prostate. The expression pattern of two proteins encoded from previously uncharacterized genes, TMEM79 and ACOXL, with elevated expression in the prostate gland were further analyzed using tissue microarrays (TMA), including normal prostate and prostate cancer, to explore their potential value as diagnostic biomarkers.

## Materials and Methods

### Tissue Samples

Fresh frozen human tissue representing 27 different normal human tissue types was included in the RNA-seq analysis as previously described [13], including 4 samples of prostatic tissue. Morphologically normal, non-cancerous prostate tissue was sampled from prostatectomy specimens derived from 4 male patients (age 62–68 y) with localized prostate cancer.

Formalin fixed, paraffin embedded (FFPE) human tissue samples were collected from the clinical Department of Pathology, Uppsala University Hospital, Uppsala, Sweden and assembled into TMAs. TMAs were created and used for protein profiling as previously described [16]. The screening TMA contained 1 mm cores of 46 different normal tissues in triplicate, including three normal prostate samples, and 216 cancer tissues representing the 20 most common cancers, including 12 cases of prostate cancer [17]. The four validation TMAs contained normal and cancerous prostate tissue; the details of each validation TMA is shown in Table 1 and have been described previously [18, 19]. Prostatic intraepithelial neoplasia was excluded from this study.

All human tissue samples used for RNA-seq and screening of protein expression were anonymized and used in accordance with approval and advisory report from the Uppsala Ethical Review Board (Reference # 2002–577, 2005–338 and 2007–159 (protein) and # 2011–473 (RNA)). The validation TMA cohorts were approved by The Central Ethical Review Board in Sweden (Dnr Ö25-2006, date 2006-06-29) and the Regional Ethical Review Board at Lund University, Sweden (approval number DN. 445–07). All patients provided written informed consent.

**Table 1:** TMA information.

TMA	Validation TMA 1	Validation TMA 2	Validation TMA 3	Validation TMA 4
**Hospital patient samples**	Uppsala University Hospital	Uppsala University Hospital	Uppsala University Hospital & Falun Central Hospital	Skåne University Hospital Malmö
**Number of prostate cases**	90	19	57	122
**Size cores and replicates**	1 mm (duplicate cores)	2 mm (1 core)	1 mm (duplicate benign cores and triplicate tumour cores)	1 mm (duplicate cores)
**Number of prostate benign cases**	15	13	57	122
**Number prostate tumour cases**	60	6	57	122
**Number prostate metastatic cases**	15	0	0	0
**Ethical approval granted by**	Research Ethics Committee at Uppsala University	Research Ethics Committee at Uppsala University	National Ethical Committee in Stockholm	Regional Ethical Review Board at Lund University
**Ethics reference code**	Ups 02–577	Ups 02–577	Dnr ö 25–2006	DN. 445–07
**Gleason grade range**	GG 2–5	GG 3–5	GG 3–5	GG 2–5
**Year range of prostatectomy samples in TMA**	1990–2006	1990–2006	1993–2001	1998–2003
**Reference previously describing TMA**	[12]	[12]	_	[13]

### Transcript profiling (RNA-seq) and data analysis

Transcriptomic profiling has been described previously [13]. Briefly, hematoxylin-eosin (HE) stained frozen sections (4 μm) were prepared from each sample using a cryostat and the CryoJane Tape-Transfer System (Instrumedics, St. Louis, MO, USA) and reviewed by a pathologist to ensure proper tissue morphology. Three 10 μm sections were cut from each frozen tissue block and homogenized prior to extraction of total RNA, using the RNeasy Mini Kit (Qiagen, Hilden, Germany) following manufacturer’s instructions. The extracted RNA samples were analyzed using either an Agilent 2100 Bioanalyzer system (Agilent Biotechnologies, Palo Alto, USA) with the RNA 6000 Nano Labchip Kit or an Experion automated electrophoresis system (Bio-Rad Laboratories, Hercules, CA, USA) with the standard-sensitivity RNA chip. Only samples of high-quality RNA (RNA Integrity Number ≥7.5) were used in the following mRNA sample preparation for sequencing. Illumina HiSeq2000 and 2500 machines (Illumina, San Diego, CA, USA) were used to perform mRNA sequencing using the standard Illumina RNA-seq protocol with a read length of 2x100 bases.

Raw reads obtained from the sequencing system were trimmed for low quality ends with the software Sickle. A phred quality threshold of 20 was used. Reads shorter than 54 bp after the trimming were discarded. The processed reads were mapped to the GRCh37 version of the human genome with Tophat v2.0.3 [20]. Potential PCR duplicates were eliminated applying the MarkDuplicates module of Picard 1.77. To obtain quantification scores for all 20,050 human protein-coding genes, FPKM (fragments per kilobase of exon model per million mapped reads) values were calculated with Cufflinks v2.0.2 [20], which corrects for transcript length and the total number of mapped reads from the library to compensate for different read depths for different samples. The average percentage of successfully mapped reads was 77%. The gene models from Ensembl build 69 [21] were used in Cufflinks. In addition to Cufflinks, HTSeq v0.5.1 was run to calculate read counts for each gene, which were used for analyses of differentially expressed genes utilizing the DESeq package [22]. All data was analyzed with R Statistical Environment [23] and a network analysis was performed using Cytoscape 3.0 [24]. For analyses performed in this study where a log2-scale of the data was used, pseudo-counts of +1 were added to the data set.

### Specificity classification

The average FPKM value in all samples for a particular tissue was used to estimate the total gene expression level. A cut-off value of 1 FPKM, roughly corresponding to an average of 1 mRNA molecule per cell, was defined as the detection limit [25]. Each of the 20,050 genes was classified into one out of nine categories based on the expression pattern in prostate in relation to all other tissues (Table 2).

**Table 2:** Specificity classification.

Category Name	Description
Not detected	-
Highly prostate-enriched	50-fold higher FPKM level in prostate compared to all other tissues
Moderately tissue-enriched	5-fold higher FPKM level in prostate compared to all other tissues
Group-enriched	5-fold higher average FPKM level within a group of 2–7 tissues including prostate compared to all other tissues
Expressed in all low	detected in 27 tissues and at least one tissue below 10 FPKM
Expressed in all high	detected in 27 tissues and all tissues above 10 FPKM
Prostate Enhanced	55-fold higher FPKM level in prostate compared to the average FPKM value of all 27 tissues
Mixed	genes expressed in 1–26 tissues and in none of the above categories

### Antibody validation: siRNA transfection, immunofluorescence, imaging and statistical analysis

Extended antibody validation to that provided on the HPA database (www.proteinatlas.org) for all proteins, was carried out to further verify the specificity of the primary antibodies to TMEM79 (HPA055214) and ACOXL (HPA035392). This was performed using siRNA-based knock-down of gene expression. U-2 OS and MCF-7 cells were used for siRNA knock-down experiments of ACOXL and TMEM79. U-2 OS cells were cultivated in McCoy’s media supplemented with 10% fetal bovine serum (FBS) and MCF-7 cells in EMEM supplemented with 10% FBS, 1% non-essential amino acids (NEAA) and 1% L-glutamine (all from FisherScientific, Stockholm, Sweden).

On the day of transfection, 10,000 U-2 OS cells or 12,000 MCF-7 cells were seeded into 96-well glass bottom plates (VWR, Stockholm, Sweden) pre-coated with fibronectin. After cell attachment, medium was replaced with 100 μl Optim-MEM containing 0,5 μl Lipofectamine 2000 (cat. No 11668019, Life Technologies) and 2,5 pmol of ACOXL siRNA (Silencer Select Pre-designed siRNA product s30651, Life Technologies) or TMEM79 siRNA (Silencer Select Pre-designed siRNA product s228349, Life Technologies). A scrambled siRNA sequence (Silencer Select Negative control no. 1, cat. no 4390843, Life Technologies) was used as negative control and AllStar (SI04381048, Qiagen) was used as a positive control to ensure successful transfection.

After 72h of incubation, cells were fixed using 4% paraformaldehyde (PFA) and permeabilized using 0.1% Triton x-100 as previously described [26]. Cells were stained with the antibodies targeting ACOXL or TMEM79, both at a concentration of 2 ng/uL. Cells were also stained with an antibody targeting the microtubules (ab7291, Abcam) at a concentration of 3 ng/μl, and with the nuclear probe 4’,6-diamidini-2-phenylindole (DAPI) at a concentration of 300 nM, to enable automated imaging and quantification of the reduced staining intensity.

The imaging and assay read-out of the siRNA experiments was done as previously described [27]. The knock-down was measured as the relative fluorescence intensity (RFI) as compared to the negative control. The data was graphically presented in box-plots and a Mann-Whitney test was used to evaluate the significance of the median RFI of the silenced cell population compared to the RFI of the corresponding negative control.

### Antibody-based tissue profiling

TMAs were cut in 4 micrometer thick sections and used for immunohistochemical staining, as previously described [16]. The immunohistochemically stained and mounted slides were scanned using an Aperio ScanScope XT Slide Scanner (Aperio Technologies, Vista, CA) for generation of high-resolution digital whole slide images, followed by annotation by certified pathologists. In brief, the manual score of IHC-based protein expression for all proteins screened was determined as the fraction of positive cells defined in different tissues: 0 = 0–1%, 1 = 2–25%, 2 = 26–75%, 3>75% and intensity of immunoreactivity: 0 = negative, 1 = weak, 2 = moderate and 3 = strong staining. All annotation and immunohistochemical data for the screening TMA together with validation data for of all primary antibodies is publically available in the Human Protein Atlas (www.proteinatlas.org) [28]. Primary antibodies used for immunostaining of validation TMAs included HPA055214 (dilution 1:250) for detection of Transmembrane protein 79 Tmem 79) and HPA035392 for detection of Acyl-CoA oxidase-like protein (ACOXL) (dilution 1:1000).

### Scoring or TMEM79 and ACOXL protein expression

The immunoreactivity of TMEM79 was assessed in the membrane and cytoplasm, and of ACOXL in the cytoplasm of epithelial cells of the prostate. Antibodies corresponding to both proteins were immunohistochemically stained and the outcome was analyzed on all validation TMAs. Scoring was performed by two independent observers (GOH, CB).

For the purpose of statistical analysis, the immunohistochemical staining pattern of both antibodies were graded according to the following scale: 0, absence of reactivity, 1, faint but clearly detectable reactivity in > 30% of epithelial cells, 2, moderate reactivity in > 30% of epithelial cells and 3, strong reactivity in > 30% of epithelial cells.

### Statistical Analysis

For statistical analysis of TMEM79 and ACOXL expression versus histopathological features, the staining intensity of the epithelial cells was divided into two groups: low expression (immunohistochemical score of 0 or 1) including cases with negative or weak staining, and high expression (immunohistochemical score of 2 or 3) including cases with moderate or strong staining. Pearson Chi square tests and Spearman and Pearson Correlation tests were performed to test the association between protein expression and histopathological features on two-way contingency tables. Diagnostic performance criteria were tested by generating ROC curves. Kaplan–Meier survival analysis and multivariate Cox regression analysis were also performed on a subset of patients, where biochemical recurrence (BCR) data was available (N = 148), to analyze the association between BCR and protein expression, serum PSA value pre-prostatectomy and Gleason score. All calculations were performed with IBM SPSS 20 for Windows (SPSS, New York, NY, USA).

## Results

### The transcriptomic analysis of prostate tissue

The transcriptomes of four prostate samples were quantified by RNA-seq and normalized mRNA levels, calculated as FPKM values [13], were determined for each sample. A total of 14,040 genes were detected in prostate, using a cutoff of mean expression value > 1 FPKM. Thus, approximately 70% of all putative protein coding genes (n = 20,050) were detected in the prostate. The distribution of FPKM values (mRNA expression levels) ranged from 0 FPKM up to 8,238, yielding a dynamic range of 10^*4*^ between the highest and lowest expressed genes. The 30 genes with the highest levels of expression in the prostate are listed in S1 Table. A majority of these genes encode for proteins with “house-keeping” functions expressed in all analyzed tissues.

**Fig 1:**
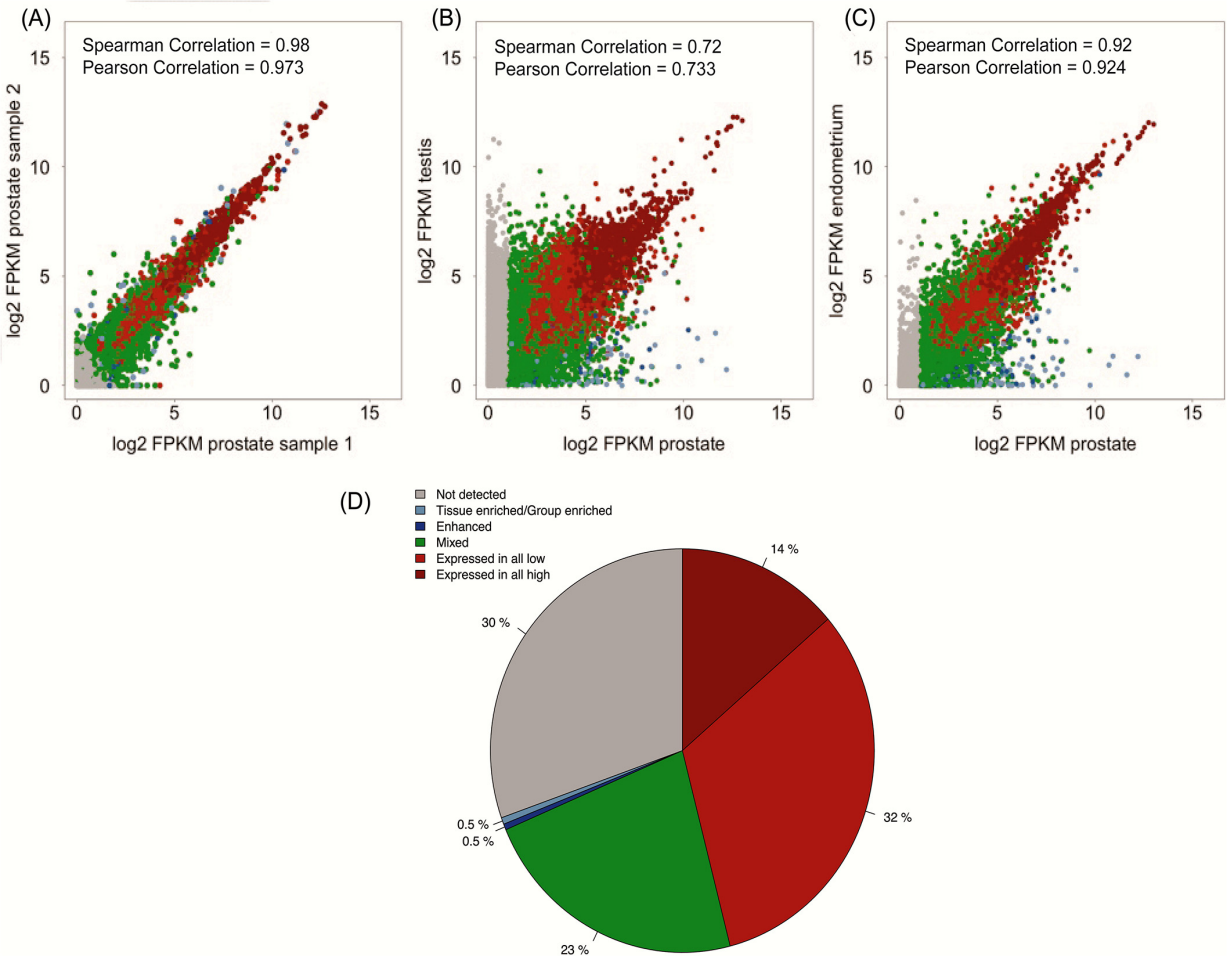
Correlation between the tissue samples and classification of all genes. Gene expression scatterplots showing all FPKM values and the pairwise Spearman and Pearson correlation coefficients between: (A) the two prostate samples with highest correlation coefficient, (B) the lowest correlation to any other tissue type (prostate vs. testis samples), and (C) the highest correlation to any other tissue type (prostate vs. endometrium). (D) Piechart showing the distribution of the fraction of all human protein-coding genes in each of the categories, based on transcript expression levels in prostate compared to all other tissues.

The biological variation between the four individual prostate samples was analyzed by comparing the expression levels of all protein coding genes in pairwise scatterplots. The correlation overall was high with Spearman coefficients ranging from 0.98 (Fig 1A) to 0.91, with an average correlation coefficient of 0.96 for all the four prostate samples. These results show low inter-individual variation across the genome-wide expression pattern and demonstrate high technical reproducibility between the prostate samples. As expected, a higher degree of variation was observed when similar comparisons were performed between prostate and other tissue types. The lowest correlation was noted between prostate and testis with a Spearman coefficient of 0.72 (Fig 1B), whereas the tissue with the highest similarity to prostate was endometrium with a Spearman coefficient of 0.92 (Fig 1C).

### Classification of the genes expressed in prostate

The transcriptomics data obtained from the 27 tissues enabled us to classify all of the 20,050 protein-coding genes into four major categories, firstly based on their expression levels in prostate (Fig 1D). These major categories included i) genes that were not detected in prostate (30%), ii) genes that showed a mixed expression pattern, being expressed in several but not in all tissue types (23%), iii) genes that were expressed in all tissues and thus characterized as “house-keeping” genes (46%) and iv) genes with an elevated level of expression in prostate as compared to other tissue types (1%). Then, the 203 genes within category iv, which showed an elevated expression pattern in prostate, were further divided into four other subcategories depending on degree of tissue specificity.

Six genes were defined as highly enriched in prostate (Table 3), characterized as the highest level of tissue-specificity with at least 50-fold higher FPKM level in prostate compared to any other tissue type. Sixteen genes were defined as moderately enriched in prostate (Table 3), with at least 5-fold higher FPKM level in prostate compared to all other tissues; 85 genes were defined as group enriched (S2 Table), with 5-fold higher average FPKM level in a group of 2–7 tissues including prostate compared to all other tissues; and 96 genes were prostate enhanced (S3 Table), defined as having a 5-fold higher FPKM level in prostate as compared to the average FPKM value of all the 27 tissues. Two well-studied genes in prostate, SLC45A3 and MSMB, were identified in this category.

**Table 3:** Highly and Moderately enriched genes in normal Prostate.

Gene name	Description	Tissue-specific score	Mean Prostate FPKM	Max FPKM in other tissue
KLK3	kallikrein-related peptidase 3	816.11	4700.79	5.76
KLK2	kallikrein-related peptidase 2	485.25	1682.84	3.47
TGM4	transglutaminase 4 (prostate)	86.13	858.59	9.97
RLN1	relaxin 1	71.53	37.05	0.52
KLK4	kallikrein-related peptidase 4	58.16	199.03	3.42
ACPP	acid phosphatase, prostate	52.20	1941.95	37.20
CHRNA2	cholinergic receptor, nicotinic, alpha 2 (neuronal)	47.78	32.15	0.67
SLC45A3	solute carrier family 45, member 3	17.65	369.97	20.96
SP8	Sp8 transcription factor	14.22	1.78	0.13
OR51E2	olfactory receptor, family 51, subfamily E, member 2	12.13	48.96	4.04
RFPL2	ret finger protein-like 2	12.04	16.53	1.37
RP11-362K2.2	Uncharacterized protein	10.80	9.79	0.91
STEAP2	STEAP family member 2, metalloreductase	10.55	136.87	12.97
OR51C1P	olfactory receptor, family 51, subfamily C, member 1 pseudogene	9.84	1.74	0.18
NKX3-1	NK3 homeobox 1	8.76	229.15	26.14
MSMB	microseminoprotein, beta-	8.59	3181.86	370.44
NEFH	neurofilament, heavy polypeptide	8.55	173.22	20.26
POTEM	POTE ankyrin domain family, member M	5.76	13.63	2.37
TRIM72	tripartite motif containing 72	5.64	2.76	0.49
RDH11	retinol dehydrogenase 11 (all-trans/9-cis/11-cis)	5.60	517.68	92.52
NCAPD3	non-SMC condensin II complex, subunit D3	5.48	127.43	23.25
LY6G6D	lymphocyte antigen 6 complex, locus G6D	5.42	3.31	0.61

**Fig 2:**
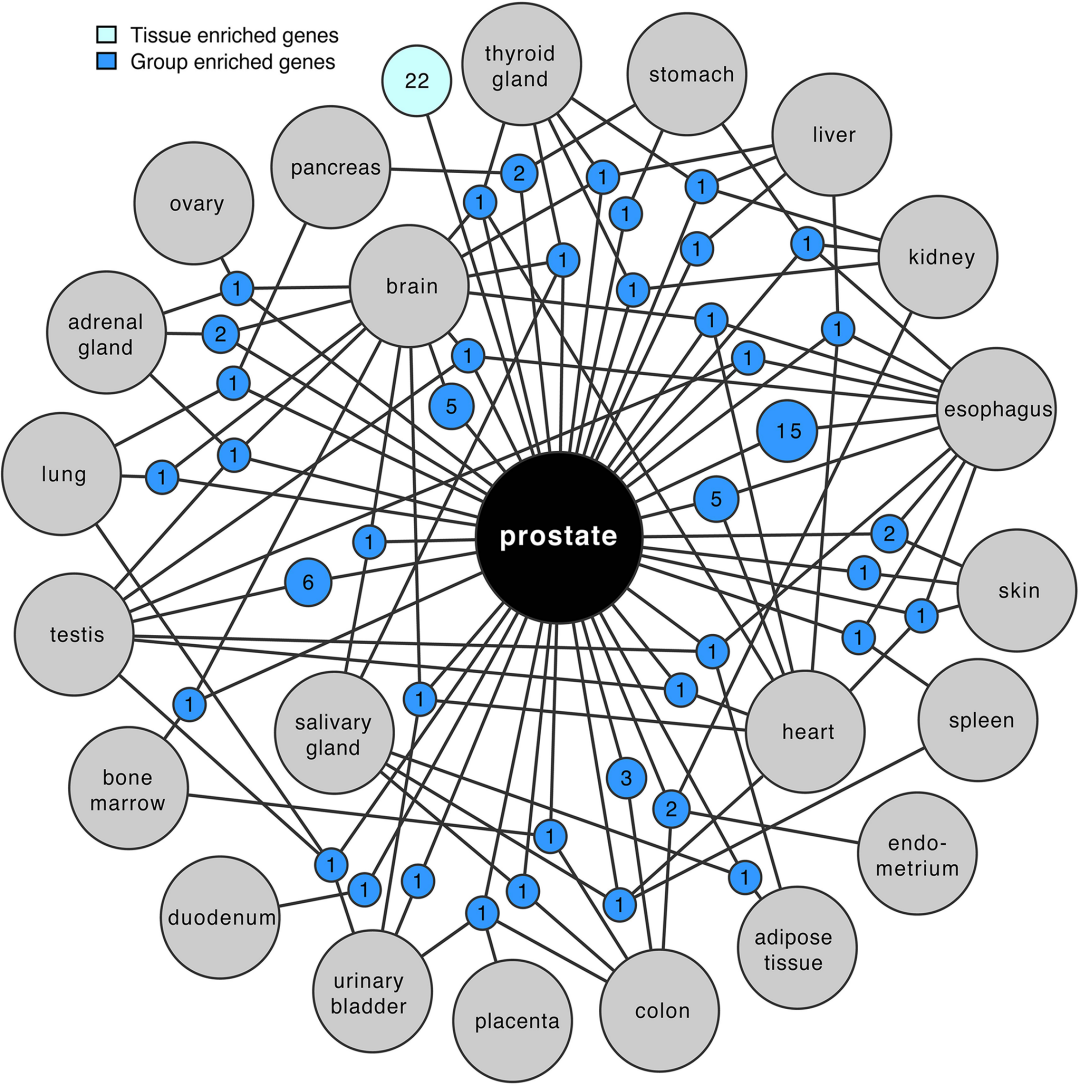
Network plot of prostate group and tissue enriched genes. Groups of expressed genes are represented as blue circle nodes and linked to the respective enriched tissues represented as grey circles. The sizes of nodes are related to the number of enriched genes. The light blue circle shows the total number of highly and moderately tissue enriched genes. The network represents an overview of the grouped enriched genes with a maximum of 4 tissues combined.

A network plot of the group-enriched genes in prostate is presented in Fig 2, which shows the number of genes shared between a particular group of tissues (up to four different tissues) as well as the number of highly and moderately tissue enriched genes in prostate. Out of the 27 tissue types analyzed, 22 tissue types had common group enriched genes with the prostate. The tissue type with most group enriched genes in common with prostate is the esophagus (n = 15), followed by testis (n = 6), brain and heart (n = 5). The shared genes between esophagus and prostate are dominated by genes expressed in various muscle cells, included in the wall of esophagus and integrated in the prostate.

### Antibody based profiling of the prostate specific genes

**Fig 3:**
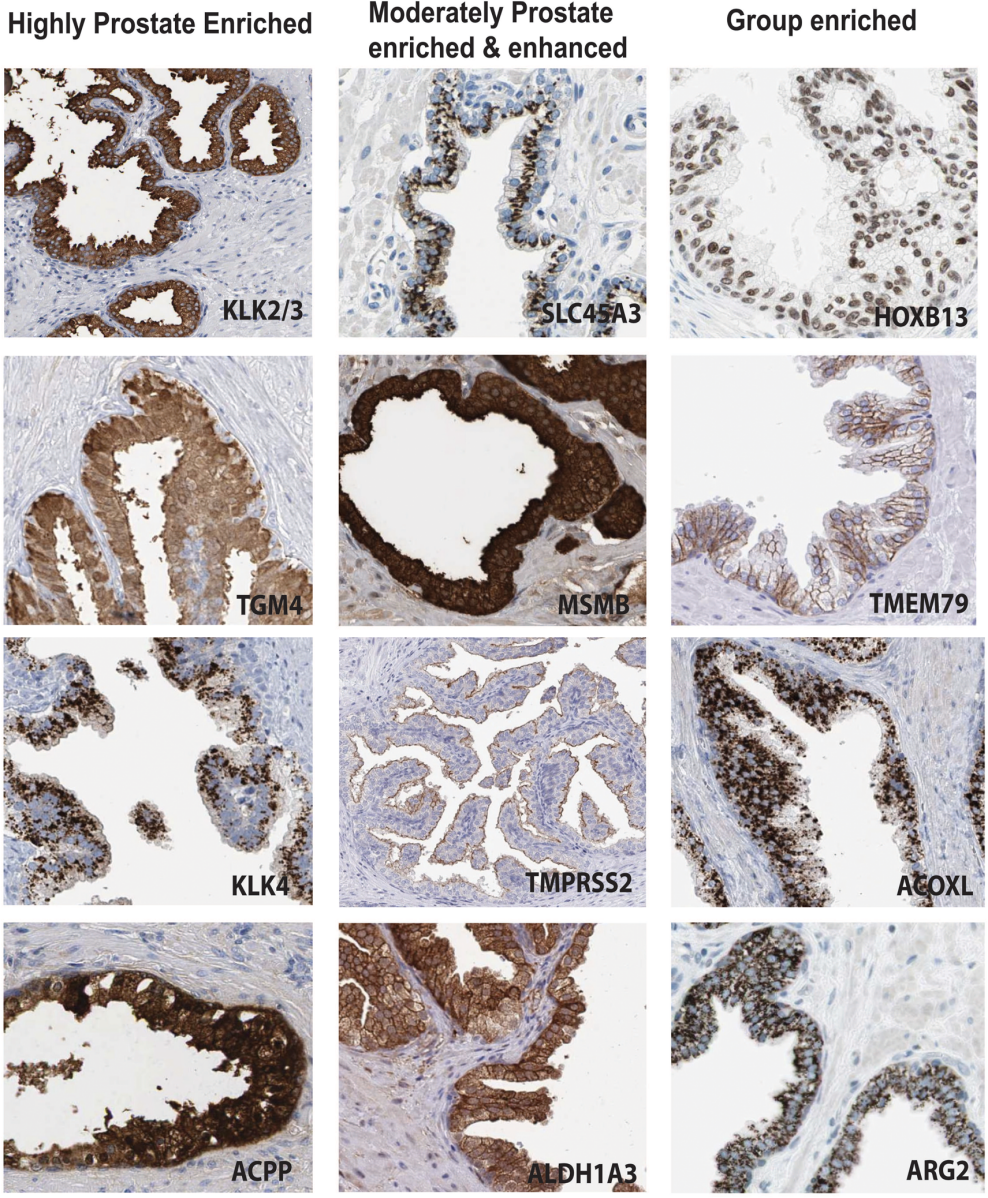
IHC-based protein expression patterns in normal prostate. Examples of expression in benign glandular cells are shown for a subset of proteins corresponding to genes with elevated expression in prostate. Scale, 100 μm.

The genes with elevated expression in prostate (n = 203) were evaluated using the online Human Protein Atlas database (HPA, www.proteinatlas.org) in order to compare the quantitative RNA-seq data with spatial expression data of corresponding protein levels. To further explore the protein expression patterns in prostate of this set of proteins, which include both previously well-studied as well as uncharacterized proteins, the immunohistochemical staining was visually evaluated with regard to benign prostate specificity and cellular distribution. Examples of expression patterns in normal prostate of proteins with elevated expression in prostate are shown in Fig 3.

### Differentially expressed proteins in benign and malignant prostatic tissue

#### Transmembrane protein 79 (TMEM79)

**Fig 4:**
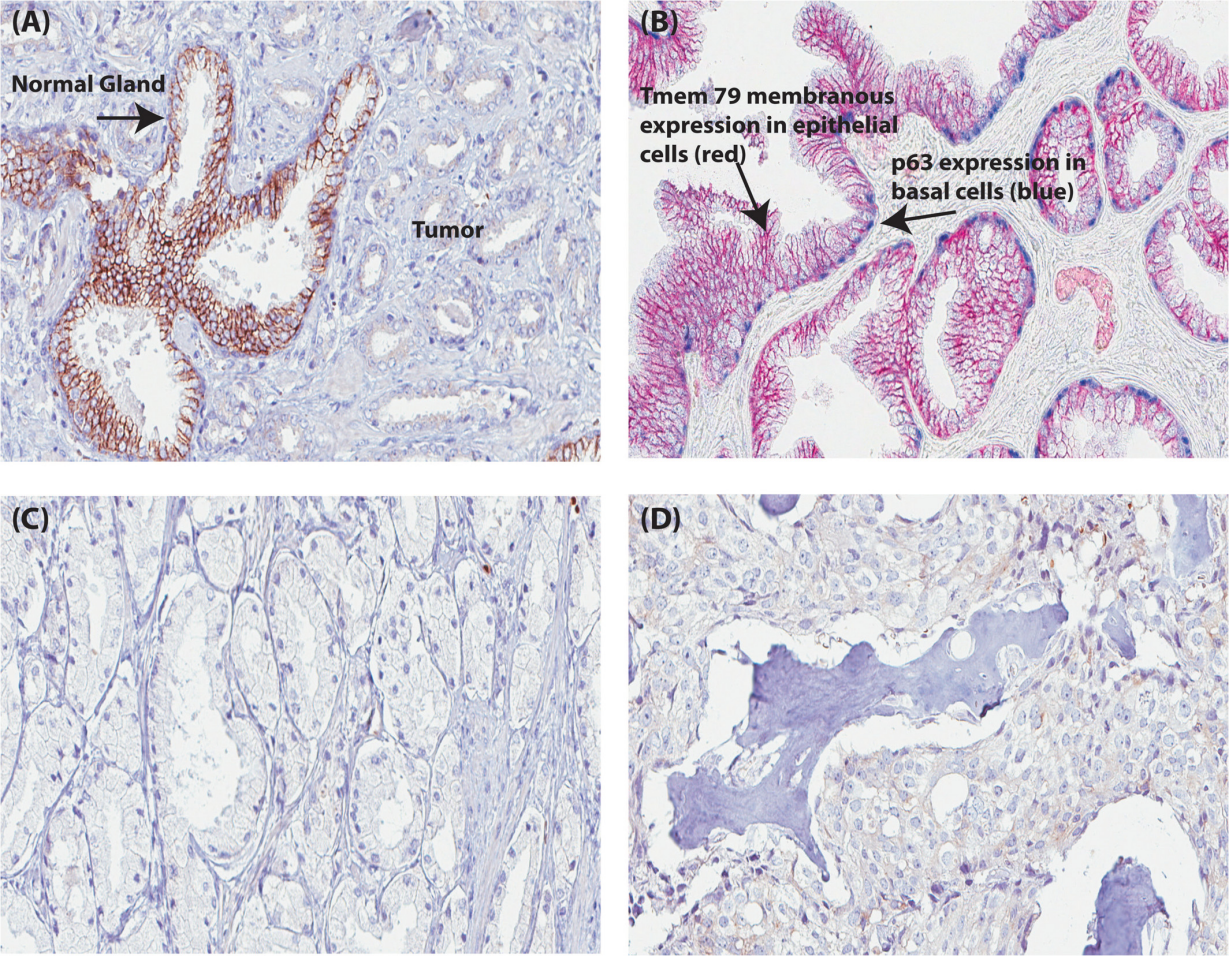
TMEM79 protein expression in prostate tissue. (A) TMEM79 membranous expression in a benign gland with lack of TMEM79 expression in surrounding tumor. (B) Dual IHC staining of TMEM79 (red) and TP63 (Blue basal cell staining) in a benign gland. (C) A lack of TMEM79 protein expression in prostate cancer (Gleason grade 3). (D) A lack of TMEM79 protein expression in prostate metastatic tumor (Bone metastasis). Scale, 100μm.

Differential protein expression in benign prostate tissue versus prostate cancer tissue was next evaluated for uncharacterized genes to which validated antibodies directed towards the corresponding proteins were available. *TMEM79*, with evidence of existence only at the transcript level according to UniProt [29], was identified as a “group enriched” gene with a FPKM value of 39 in prostate. TMEM79 gene expression was also observed in esophagus (54 FPKM) and skin (65 FPKM) at higher levels than in prostate (see S2 Table). However, at the protein level TMEM79 showed a more distinct immunoreactivity in normal prostate glands compared with the expression pattern in squamous epithelia in skin, esophagus, oral mucosa, vagina and cervix. Strong membranous immunostaining was observed in 3/3 benign prostate tissue cases, whereas there was no evident membranous staining in tumor cells from 12/12 cases of prostate cancer (images of immunostained normal and cancerous prostate tissues are available at www.proteinatlas.org and in Fig 4).

To validate the initial protein screening results observed for TMEM79 in prostate, IHC was performed on four independent prostate cancer cohorts. Tissues from 333 cases were available for analysis (156 benign cases, 162 primary prostate cancer cases and 15 metastatic prostate cancer cases). Approximately 81% (127/156) of benign prostate tissue samples showed a positive membranous expression of TMEM79 and approximately 84% (148/156) of prostate cancer tissue samples did not express TMEM79 (Table 4). Thus, TMEM79 displayed a high sensitivity (81%) and specificity (84%) to distinguish benign prostate glands from prostate cancer. To statistically assess the hypothesis that positive TMEM79 expression is inversely associated to prostate cancer, a two-way contingency table was set up (Table 4), which classified the test variables into categories; benign tissue versus tumor (Gleason grade 2–5 and metastasis). To statistically assess the diagnostic performance criteria of TMEM79 a ROC curve was generated (S1 Fig) which produced an AUC value of 0.825 and a significant *P value* of 0.000 indicating that TMEM79 is a good diagnostic test for distinguishing between benign glands and tumor of the prostate. No association between BCR, serum PSA value pre-prostatectomy, Gleason score and TMEM79 expression was observed by either Kaplan–Meier survival analysis or multivariate Cox regression analysis on the subset of 148 patients analyzed.

**Table 4:** Statistical analysis for TMEM79 Protein expression.

Tmem79 Membranous Expression vs. Histology (Gleason grade)	Benign	Gleason grade 2	Gleason grade 3	Gleason grade 4	Gleason grade 5	Metastasis	Total
**Negative**	29 (18.6%)	3 (100.0%)	59 (77.6%)	55 (88.7%)	19 (90.5%)	12 (80.0%)	**177**
**Positive**	127 (81.4%)	0 (0.0%)	17 (22.4%)	7 (11.3%)	2 (9.5%)	3 (20.0%)	**156**
**Total**	**156**	**3**	**76**	**62**	**21**	**15**	**333**
**Tmem79 Membranous Expression vs. Histology**	**Benign**	**Tumor & Metastasis**	**Total**	**Chi Square value**	140.80	***P value***	*0*.*000*
**Negative**	29 (18.6%)	148 (83.6%)	**177**	**Likelihood Ratio**	152.60	***P value***	*0*.*000*
**Positive**	127 (81.4%)	29 (16.4%)	**156**	**Pearson's R**	-0.65	***P value***	*0*.*000*
**Total**	**156**	**177**	**333**	**Spearman's Correl.**	-0.65	***P value***	*0*.*000*
**Membranous vs. Cytoplasmic expression**	** **	**Cytoplasmic Expression**	** **	**Chi Square value**	19.39	***P value***	*0*.*000*
** **		**Negative**	**Positive**	**Likelihood Ratio**	19.94	***P value***	*0*.*000*
**Membranous**	**Negative**	105 (18.6%)	73 (83.6%)	**Pearson's R**	-0.24	***P value***	*0*.*000*
**Expression**	**Positive**	126 (81.4%)	29 (16.4%)	**Spearman's Correl.**	-0.24	***P value***	*0*.*000*

#### Acyl-CoA oxidase-like protein (ACOXL)

Similar to TMEM79, ACOXL was identified as a novel tissue marker of benign prostate following screening of protein expression in the Human Protein Atlas. In contrast to the membranous expression of TMEM79, ACOXL showed a strong granular, cytoplasmic expression pattern in benign prostatic glands. *ACOXL* was identified as a “group enriched” gene also with a FPKM value of 3 in prostate tissue. Other tissues that were classified as “group enriched” for the ACOXL gene were lung (15 FPKM), urinary bladder (13 FPKM) and testis (4 FPKM). Immunostaining of ACOXL protein showed a strong granular, cytoplasmic staining also in bronchi and the fallopian tube as well as a weaker and diffuse staining pattern in lung, skin, gallbladder, salivary gland, thyroid, adrenal gland, vagina and brain.

**Fig 5:**
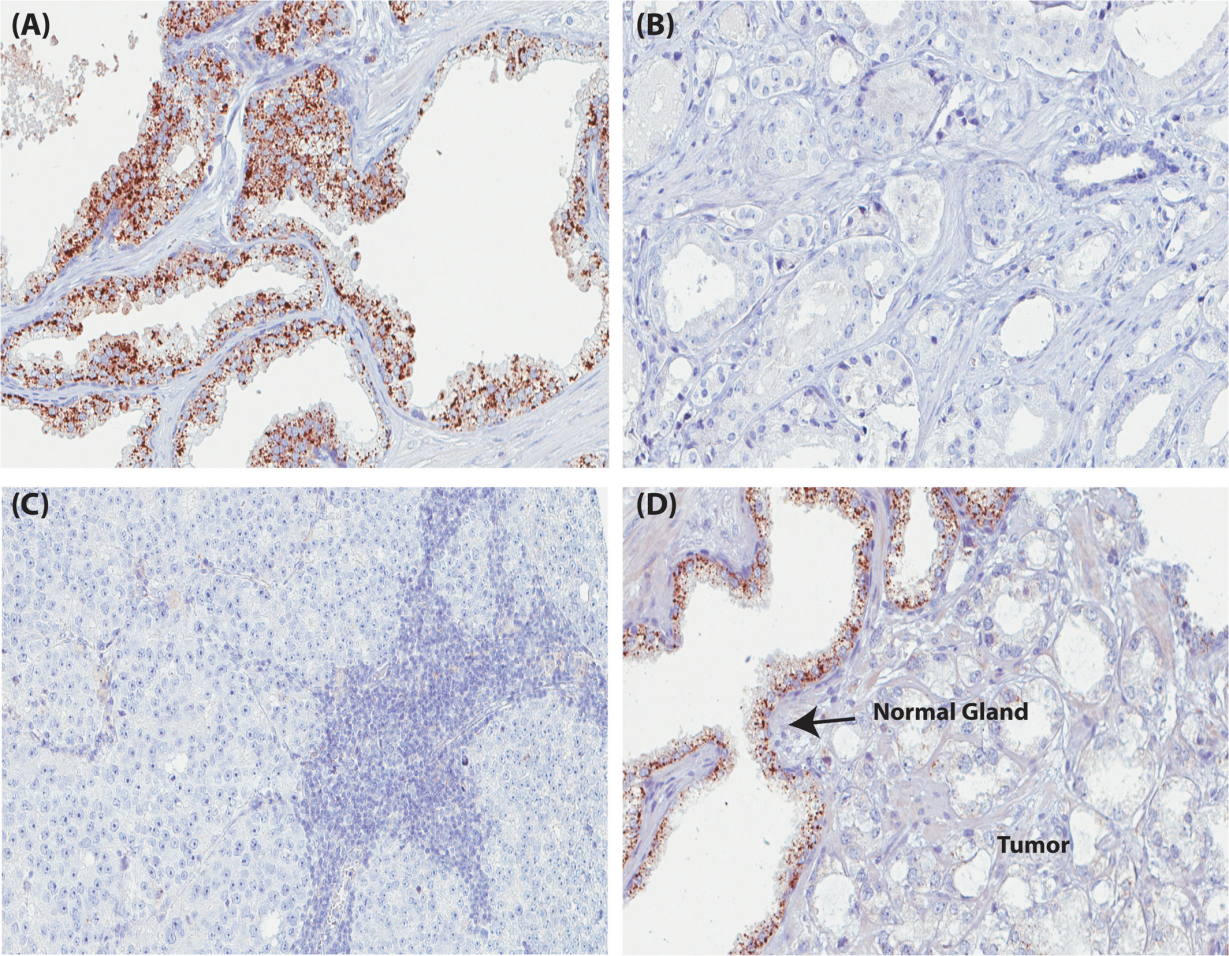
ACOXL protein expression in prostate tissue. (A) Granular, cytoplasmic expression of ACOXL in a benign prostatic hyperplasia. (B) A lack of ACOXL protein expression in prostate cancer (Gleason grade 3). (C) A lack of ACOXL protein expression in prostate metastatic tumor (lymph node metastasis). (D) Granular, cytoplamic expression of ACOXL in a benign gland with lack of TMEM79 expression in surrounding tumor. Scale, 100μm.

3/3 benign prostate tissue cases showed a positive staining for ACOXL expression and no staining was observed in 8/12 prostate cancers (see Fig 5 and www.proteinatlas.org for images of immunostained normal and cancerous prostate).

IHC was further performed on the four independent prostate cancer cohorts. Approximately 86% (132/154) benign prostate tissue samples had positive cytoplasmic ACOXL expression and approximately 72% (129/179) prostate cancer tissue samples did not express ACOXL, suggesting a high specificity and sensitivity also for ACOXL to identify benign prostate glands.

A similar contingency table and statistical analyses as was done for TMEM79 was performed on the ACOXL data (Table 5). Both Pearson and Spearman correlations also showed that there is a moderate inverse relationship between the membranous expression of TMEM79 and prostate cancer. Separating the tumor category into Gleason grades and metastatic tumors showed a trend for decreased ACOXL expression in more advanced and aggressive tumors. No association between BCR and ACOXL expression in prostate cancer tissue was observed. To statistically assess the diagnostic performance criteria of ACOXL a ROC curve was generated (S1 Fig) which produced an AUC value of 0.788 and a significant *P value* of 0.000 indicating that ACOXL is a good diagnostic test for distinguishing between benign glands and tumor of the prostate. No association between BCR, serum PSA value pre-prostatectomy, Gleason score and ACOXL expression was observed by either Kaplan–Meier survival analysis or multivariate Cox regression analysis on the subset of 148 patients analyzed.

**Table 5:** Statistical analysis for ACOXL Protein expression.

Acoxl Expression vs. Histology (Gleason grade)	Benign	Gleason grade 2	Gleason grade 3	Gleason grade 4	Gleason grade 5	Metastasis	Total
**Negative**	22 (14.3%)	2 (50.0%)	50 (65.8%)	48 (75.0%)	16 (80.0%)	13 (80.0%)	**177**
**Positive**	132 (85.7%)	2 (50.0%)	26 (34.2%)	16 (25.0%)	4 (20.0%)	2 (20.0%)	**156**
**Total**	**154**	**4**	**76**	**64**	**20**	**15**	**333**
**Acoxl Expression vs. Histology**	**Benign**	**Tumor & Metastasis**	**Total**	**Chi Square value**	111.50	***P value***	*0*.*000*
**Negative**	22 (14.3%)	129 (72.1%)	**151**	**Likelihood Ratio**	120.40	***P value***	*0*.*000*
**Positive**	132 (85.7%)	50 (27.9%)	**182**	**Pearson's R**	-0.58	***P value***	*0*.*000*
**Total**	**154**	**179**	**333**	**Spearman's Correlation**	-0.58	***P value***	*0*.*000*

### Antibody validation for Rabbit Polyclonal anti-TMEM79 (HPA 055214) and Rabbit Polyclonal anti-ACOXL (HPA035392)

To evaluate the specificity of the primary antibodies targeting ACOXL (HPA035392) and TMEM79 (HPA055214), the target proteins were knocked down using siRNA in U-2 OS and MCF-7 cells, and analyzed using immunofluorescence (S2 Fig). The immunofluorescence staining of ACOXL showed a mainly cytoplasmic staining pattern that was significantly decreased after silencing of the corresponding transcript in both U-2 OS and MCF-7 cells, indicating a specific binding of the ACOXL antibody to the intended target protein with a RFI of 58 and 70% respectively (S2 Fig).

For TMEM79, a significant decrease in staining intensity was observed in both cell lines (RFI of 74 and 70% for U-2 OS and MCF-7), also indicating a specific binding of the antibody to the TMEM79 protein (S2 Fig). In addition to staining in the cell membrane, as seen with IHC, immunofluorescence staining was found in the nucleoli. Additional image analysis of the nuclear staining intensity, showed a slight decrease in the silenced cells compared to the controls (data not shown). However, this decrease was not significant.

## Discussion

Current clinical biomarkers for prostate cancer lack specificity and sensitivity to reliably distinguish aggressive versus non-aggressive prostate cancer [2–5]. Thus, determining which patients require treatment and stratification of patients that benefit from aggressive treatment strategies remains a diagnostic and clinical dilemma.

While major advancements in proteomic and metabolomic research have been seen in the past decade producing potential biomarkers for cancer, most have failed to replace existing markers due to lack of added value [30]. A concrete approach to discover and identify specific biomarkers is to search for proteins that are specifically expressed in the tissue of interest prior to searching for such discriminating proteins in the blood or urine [31].

Unlike other previously published tissue specific expression studies, we combined a state of the art RNA-seq data set describing the prostate specific transcriptome with corresponding *in situ* protein expression in benign prostate. Our RNA-seq analysis identified 6 highly enriched genes in prostate, 16 moderately enriched genes, 85 group enriched genes and 96 prostate enhanced genes. Several genes with elevated expression in prostate (Table 3 and S2 and S3 Tables) are previously well-known genes in prostate cancer, and include *KLK3* (Prostate Specific Antigen), which had the highest FPKM value (4701) of all genes with elevated expression in prostate, and *ACPP* (Human Prostatic Acid Phosphatase) which had a FPKM value of 1942, [32–34]. Other genes identified as highly enriched in prostate cancer were *TGM4*, *KLK2* and *KLK4*, the latter kalekrein-related proteins belonging to the same family as PSA (KLK3). All three genes/proteins have been well characterized in prostate tissue [35]. The identification of these genes in this category validates the RNA-seq and antibody profiling approach taken in our study to identifying prostate tissue specific markers.

The most interesting finding in this study was the identification of two novel, uncharacterized genes in prostate from the “group enriched” category, *TMEM79* and *ACOXL*. Both genes showed excellent protein and RNA correlation and differential protein expression in benign and prostate tissue and, thus, were chosen to be further evaluated on a larger cohort of prostate cancer cases to investigate if they would be good markers of benign prostate in tissue.

*TMEM79*, which encodes transmembrane protein 79, is a member of the transmembrane protein (TMP) family which plays a crucial role in cells acting primarily as transporters and receptors [36]. TMPs and misassembly of these proteins are related to several serious diseases. For example, the I655V mutation in the transmembrane α-helix of *ERBB2* has been shown to increase risk of breast cancer and cystic fibrosis has been attributed to endoplasmic reticulum defects caused by misassembly of CFTR (the cystic fibrosis transmembrane conductance regulator) [37], making TMPs biological drug targets [38]. In our study, we report strong membranous protein expression of TMEM79 in approximately 82% of benign prostate glands and a lack of membranous TMEM79 expression in 84% of prostate tumors, corresponding to approximately 82% sensitivity and 84% specificity at identifying benign prostate glands in tissue. In some cases of tumor where membranous expression was lost weak cytoplasmic expression was observed. This may indicate a translocation of the protein from the membrane internally to the cell cytoplasm in the conversion of normal epithelium to tumor epithelium. The underlying mechanisms and potential role of loss of TMEM79 expression in prostate cancer cells are unknown as the function of TMEM79 has yet to be elucidated. It could be speculated that a possible mutation or defect such as a deletion in the gene may play a role in its loss of expression as like many members of the TMP family. However, future functional studies of TMEM79 and further sequencing of prostate tumor tissue will be important to increase our knowledge regarding this promising prostate cancer marker.

Acyl-Coenzyme A oxidase-like (ACOXL) is proposed to participate in fatty acid β-oxidation, fatty acid metabolic process and oxidation reduction according to NCBI”Aceview” [39][39](39). ACOXL displayed strong granular cytoplasmic expression in mitochondrial regions in approximately 86% of benign glands and this expression was lost in 72% of prostate tumors, corresponding to approximately 86% sensitivity and 72% specificity in identifying benign prostate glands in tissue. An increasing trend of ACOXL protein expression loss in more poorly differentiated and aggressive tumors was also noted. Recently, an enrichment of the *ACOXL* gene has been implicated in prostate cancer serum using metabolic quantitative trait loci analysis in the serum of 402 Swedish men [40]. This could suggest that the transformation in phenotype of normal epithelium to tumor epithelium may result in loss of ACOXL protein expression in the epithelium potentially due to leakage of ACOXL into the serum. However, further analysis of protein levels in both tissue and matched serum is warranted to further evaluate this hypothesis.

Our study revealed the identification of two potential, novel biomarkers of benign prostate, TMEM79 and ACOXL. We observed high sensitivity and specificity of these markers at detecting benign prostate which may suggest that these markers could be beneficial at detecting benign tissue on biopsy to assist pathological diagnosis of benign glands in combination with other basal cell markers such as p63. Although these markers do not show as high sensitivity and specificity as other diagnostic IHC basal cell markers such as TP63 or antibodies detecting high molecular weight cytokeratins (34βE12) at identifying benign glands, we have shown that these markers are specific for benign prostate epithelium and speculate that based on previously reported serum analysis in combination with our findings that ACOXL could be leaked into serum during tumor growth, yielding a potential marker for prostate cancer screening. However, further analysis of TMEM79 and ACOXL at a number of levels including functional analysis, analysis of ACOXL expression levels in matched tissue and serum are warranted in prostate cancer tissue in order to determine if they have clinical impact in disease screening.

In conclusion, our model of RNA-seq analysis and immunohistochemistry-based protein profiling in human normal tissues provides an advantageous strategy to identify tissue specific markers of diseases such as prostate cancer. Using this strategy TMEM79 and ACOXL were identified as two novel candidate biomarkers for prostate cancer.

## Supporting Information

S1 Fig Receiver Operating Characteristic (ROC) curve analysis (A) ROC curve testing the diagnostic performance criteria of TMEM79 where an AUC of 0.825 is observed (B) ROC curve testing the diagnostic performance criteria of TMEM79 where an AUC of 0.788 is observed. (TIF)

S2 Fig Antibody validation of TMEM79 (HPA035392) and ACOXL (HPA055214) by siRNA gene knock down and immunofluorescence. (A) ACOXL immunofluorescence staining of U-2 OS cells with *ACOXL* siRNA gene knock down and U-2 OS control cells. (B) ACOXL immunofluorescence staining of MCF-7 cells with *ACOXL* siRNA gene knock down and MCF-7 control cells. (C) TMEM79 immunofluorescence staining of U-2 OS cells with *TMEM79* siRNA gene knock down and U-2 OS control cells. (D) TMEM79 immunofluorescence staining of MCF-7 cells with *TMEM79* siRNA gene knock down and MCF-7 control cells.
(TIF)

S1 Table The top 30 genes with the highest levels of expression in the prostate. (DOCX)

S2 Table Group enriched genes which were defined as having at least 5-fold higher FPKM level in a group of 2–7 tissues including prostate compared to all other tissues. (DOCX)

S3 Table Enhanced genes in the Prostate defined as having a 5-fold higher FPKM level in prostate as compared to the average FPKM value of all the 27 tissues. (DOCX)
