# Supplementary figures and images for: Analysis of the Human Prostate-Specific Proteome Defined by Transcriptomics and Antibody-Based Profiling Identifies TMEM79 and ACOXL as Two Putative, Diagnostic Markers in Prostate Cancer
Source: PLoS One. 2015 Aug 3;10(8):e0133449. doi: 10.1371/journal.pone.0133449 (PMC4523174; doi:10.1371/journal.pone.0133449)

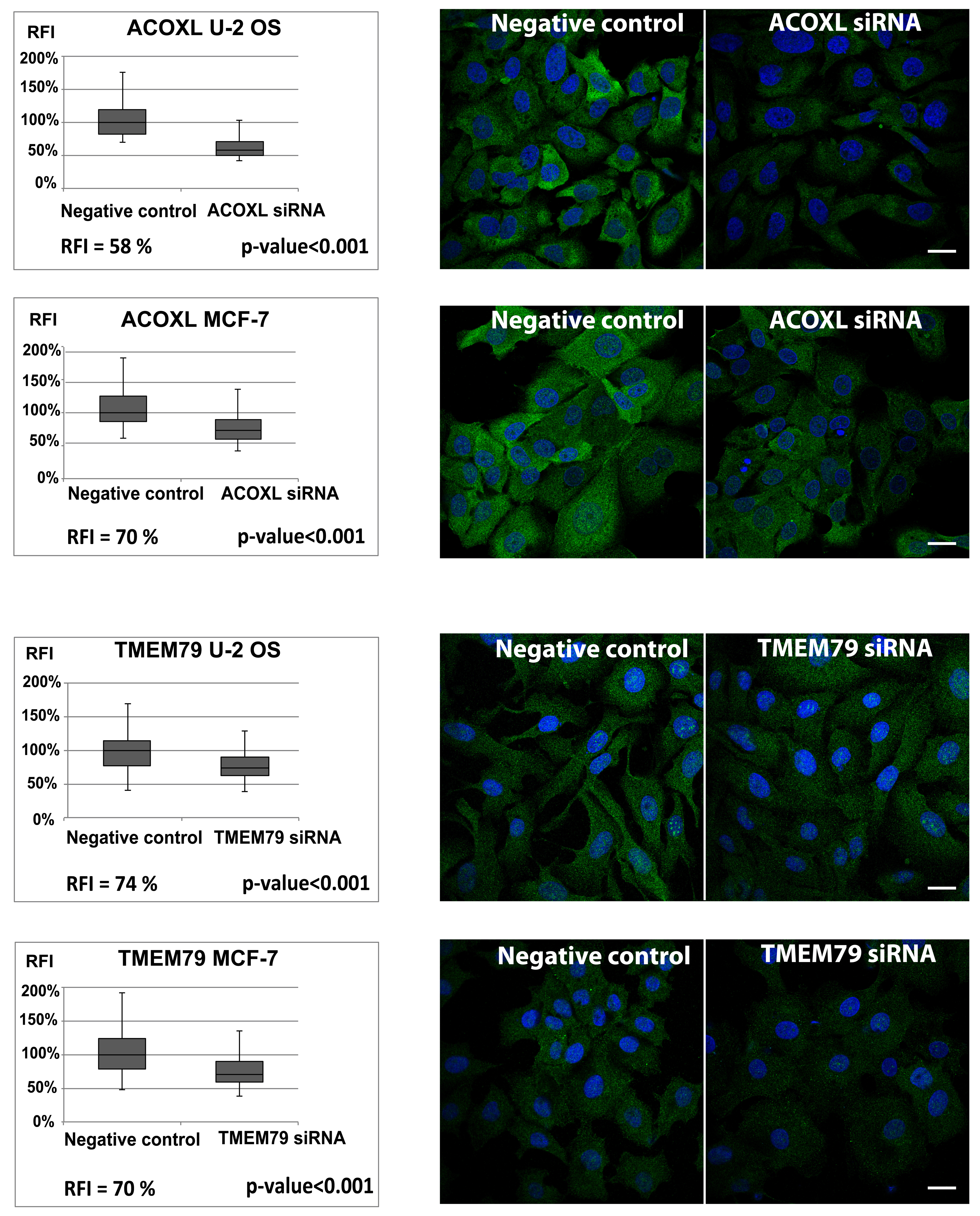

Supplement: S2 Fig — (A) ACOXL immunofluorescence staining of U-2 OS cells with ACOXL siRNA gene knock down and U-2 OS control cells. (B) ACOXL immunofluorescence staining of MCF-7 cells with ACOXL siRNA gene knock down and MCF-7 control cells. (C) TMEM79 immunofluorescence staining of U-2 OS cells with TMEM79 siRNA gene knock down and U-2 OS control cells. (D) TMEM79 immunofluorescence staining of MCF-7 cells with TMEM79 siRNA gene knock down and MCF-7 control cells. (TIF) [file pone.0133449.s002.tif]
