# Supplementary material for: Analysis of the Human Prostate-Specific Proteome Defined by Transcriptomics and Antibody-Based Profiling Identifies TMEM79 and ACOXL as Two Putative, Diagnostic Markers in Prostate Cancer
Source: PLoS One. 2015 Aug 3;10(8):e0133449. doi: 10.1371/journal.pone.0133449 (PMC4523174; doi:10.1371/journal.pone.0133449)
Supplement: S1 Table — (DOCX) [file pone.0133449.s003.docx]

**Supplementary Table 1: The top 30 genes with the highest levels of expression in the prostate**

| **Gene name** | **Description** | **Number of tissues that gene is detected in** | **Mean Prostate FPKM** |
| --- | --- | --- | --- |
| MT-CO1 | mitochondrially encoded cytochrome c oxidase I | 27 | 8238.1 |
| MT-ATP8 | mitochondrially encoded ATP synthase 8 | 27 | 6885.4 |
| KLK3 | kallikrein-related peptidase 3 | 15 | 5955.0 |
| MT-CO3 | mitochondrially encoded cytochrome c oxidase III | 27 | 5494.1 |
| MT-ATP6 | mitochondrially encoded ATP synthase 6 | 27 | 5045.9 |
| MT-CO2 | mitochondrially encoded cytochrome c oxidase II | 12 | 4700.8 |
| MT-ND4 | mitochondrially encoded NADH dehydrogenase 4 | 27 | 4689.9 |
| MT-ND3 | mitochondrially encoded NADH dehydrogenase 3 | 27 | 3503.3 |
| MT-CYB | mitochondrially encoded cytochrome b | 27 | 3413.3 |
| MT-ND4L | mitochondrially encoded NADH dehydrogenase 4L | 27 | 3336.0 |
| KLK2 | kallikrein-related peptidase 2 | 13 | 3302.2 |
| MSMB | microseminoprotein, beta- | 17 | 3181.9 |
| B2M | beta-2-microglobulin | 27 | 3037.3 |
| MT-ND2 | mitochondrially encoded NADH dehydrogenase 2 | 27 | 2686.1 |
| MT-ND6 | mitochondrially encoded NADH dehydrogenase 6 | 27 | 2402.2 |
| MT-ND1 | mitochondrially encoded NADH dehydrogenase 1 | 27 | 2228.0 |
| MT-ND5 | mitochondrially encoded NADH dehydrogenase 5 | 27 | 1953.6 |
| TAGLN | transgelin | 24 | 1941.9 |
| ACPP | acid phosphatase, prostate | 23 | 1682.8 |
| ACTA2 | actin, alpha 2, smooth muscle, aorta | 27 | 1444.5 |
| EEF1A1 | eukaryotic translation elongation factor 1 alpha 1 | 27 | 1350.7 |
| TPT1 | tumor protein, translationally-controlled 1 | 27 | 1330.1 |
| RPL37A | ribosomal protein L37a | 27 | 1321.7 |
| DES | desmin | 27 | 1212.8 |
| ACTG2 | actin, gamma 2, smooth muscle, enteric | 27 | 1163.1 |
| ACTB | actin, beta | 27 | 1150.6 |
| MYL6 | myosin, light chain 6, alkali, smooth muscle and non-muscle | 27 | 1093.8 |
| RPS24 | ribosomal protein S24 | 27 | 1088.8 |
| MYL9 | myosin, light chain 9, regulatory | 27 | 1061.0 |
| CLU | clusterin |  |  |
