# Supplementary material for: Analysis of the Human Prostate-Specific Proteome Defined by Transcriptomics and Antibody-Based Profiling Identifies TMEM79 and ACOXL as Two Putative, Diagnostic Markers in Prostate Cancer
Source: PLoS One. 2015 Aug 3;10(8):e0133449. doi: 10.1371/journal.pone.0133449 (PMC4523174; doi:10.1371/journal.pone.0133449)
Supplement: S2 Table — (DOCX) [file pone.0133449.s004.docx]

**Supplementary Table 2: Group enriched genes which were defined as having** **at least 5-fold higher FPKM level in a group of 2-7 tissues including prostate compared to all other tissues**

| **Gene name** | **Description** | **Group Specific Tissues** | **Mean Prostate FPKM** | **Max FPKM in other tissue** | **Tissue-specific score** |
| --- | --- | --- | --- | --- | --- |
| POTEG | POTE ankyrin domain family, member G | prostate, testis | 12.31 | 2.49 | 4.95 |
| POTEH | POTE ankyrin domain family, member H | prostate, testis | 6.01 | 1.65 | 3.64 |
| NPY | neuropeptide Y | prostate, brain, adrenal | 323.81 | 93.02 | 3.48 |
| MYBPC2 | myosin binding protein C, fast type | prostate, esophagus | 12.78 | 4.15 | 3.08 |
| TTC6 | tetratricopeptide repeat domain 6 | prostate, testis | 16.01 | 6.05 | 2.65 |
| LMAN1L | lectin, mannose-binding, 1 like | prostate, spleen, heart, salivary gland | 76.80 | 33.10 | 2.32 |
| TRPM8 | transient receptor potential cation channel, subfamily M, member 8 | liver, prostate | 111.80 | 49.08 | 2.28 |
| MYOG | myogenin (myogenic factor 4) | prostate, esophagus | 3.74 | 1.74 | 2.15 |
| EYA1 | eyes absent homolog 1 (Drosophila) | prostate, brain, urinary bladder, heart | 12.68 | 6.29 | 2.02 |
| HOXB13 | homeobox B13 | colon, prostate | 65.44 | 33.58 | 1.95 |
| MYBPC1 | myosin binding protein C, slow type | prostate, esophagus | 154.79 | 93.70 | 1.65 |
| TMOD4 | tropomodulin 4 (muscle) | prostate, esophagus | 17.48 | 11.97 | 1.46 |
| MYF6 | myogenic factor 6 (herculin) | prostate, esophagus | 7.68 | 5.27 | 1.46 |
| MYH3 | myosin, heavy chain 3, skeletal muscle, embryonic | prostate, testis, esophagus | 33.07 | 22.77 | 1.45 |
| MYL1 | myosin, light chain 1, alkali; skeletal, fast | prostate, esophagus | 250.64 | 182.09 | 1.38 |
| KBTBD5 | kelch repeat and BTB (POZ) domain containing 5 | prostate, esophagus | 5.48 | 4.08 | 1.35 |
| TMEFF2 | transmembrane protein with EGF-like and two follistatin-like domains 2 | prostate, brain | 33.25 | 24.95 | 1.33 |
| HOXA13 | homeobox A13 | colon, prostate, placenta, urinar ybladder | 32.62 | 24.73 | 1.32 |
| UPK3A | uroplakin 3A | prostate, urinary bladder | 15.33 | 11.68 | 1.31 |
| CHRM1 | cholinergic receptor, muscarinic 1 | prostate, brain, salivary gland | 23.93 | 19.78 | 1.21 |
| MYLPF | myosin light chain, phosphorylatable, fast skeletal muscle | prostate, esophagus | 68.64 | 56.88 | 1.21 |
| LRRC26 | leucine rich repeat containing 26 | colon, prostate, salivary gland | 27.98 | 23.56 | 1.19 |
| PRAC | prostate cancer susceptibility candidate | colon, prostate | 113.73 | 98.23 | 1.16 |
| KBTBD10 | kelch repeat and BTB (POZ) domain containing 10 | prostate, esophagus, heart | 89.95 | 77.71 | 1.16 |
| MYH2 | myosin, heavy chain 2, skeletal muscle, adult | prostate, esophagus | 46.06 | 41.64 | 1.11 |
| TNNI1 | troponin I type 1 (skeletal, slow) | prostate, esophagus | 102.87 | 101.23 | 1.02 |
| DLX1 | distal-less homeobox 1 | prostate, brain, adrenal | 3.23 | 3.37 | 0.96 |
| SPOCK3 | sparc/osteonectin, cwcv and kazal-like domains proteoglycan (testican) 3 | prostate, brain, adrenal, ovary | 67.35 | 71.79 | 0.94 |
| MYH1 | myosin, heavy chain 1, skeletal muscle, adult | prostate, fat, testis, esophagus | 3.63 | 3.90 | 0.93 |
| MYOT | myotilin | prostate, esophagus, heart | 36.68 | 39.56 | 0.93 |
| HOXD13 | homeobox D13 | colon, prostate | 10.33 | 11.30 | 0.91 |
| PLA2G4D | phospholipase A2, group IVD (cytosolic) | prostate, skin | 11.39 | 13.18 | 0.86 |
| CUX2 | cut-like homeobox 2 | liver, prostate, brain, thyroid | 9.88 | 11.49 | 0.86 |
| ARG2 | arginase, type II | kidney, prostate, thyroid | 91.77 | 112.92 | 0.81 |
| SORD | sorbitol dehydrogenase | kidney, liver, prostate, thyroid | 195.37 | 243.16 | 0.80 |
| TNNC2 | troponin C type 2 (fast) | prostate, esophagus | 75.93 | 96.09 | 0.79 |
| CACNA1S | calcium channel, voltage-dependent, L type, alpha 1S subunit | prostate, esophagus | 2.33 | 2.98 | 0.78 |
| TNNT1 | troponin T type 1 (skeletal, slow) | prostate, esophagus, heart | 265.72 | 373.30 | 0.71 |
| PABPC1L2B | poly(A) binding protein, cytoplasmic 1-like 2B | prostate, brain, adrenal, testis | 3.15 | 4.53 | 0.69 |
| CASQ1 | calsequestrin 1 (fast-twitch, skeletal muscle) | prostate, brain, esophagus, heart | 27.52 | 40.64 | 0.68 |
| TNNT3 | troponin T type 3 (skeletal, fast) | prostate, fat, placenta, urinary bladder, esophagus, heart | 56.56 | 84.20 | 0.67 |
| AC120194.1 |  | prostate, lymphnode, adrenal, heart, skin, bonemarrow, salivarygland | 10.31 | 15.42 | 0.67 |
| CACNG1 | calcium channel, voltage-dependent, gamma subunit 1 | prostate, esophagus | 2.84 | 4.31 | 0.66 |
| TRPV6 | transient receptor potential cation channel, subfamily V, member 6 | pancreas, prostate, duodenum, placenta, gallbladder, skin, salivary gland | 42.68 | 68.59 | 0.62 |
| CECR6 | cat eye syndrome chromosome region, candidate 6 | prostate,brain | 5.15 | 8.52 | 0.60 |
| TMEM79 | transmembrane protein 79 | prostate, esophagus, skin | 38.59 | 64.78 | 0.60 |
| FBXL22 | F-box and leucine-rich repeat protein 22 | prostate, cervix, gallbladder, urinary bladder, esophagus, heart | 14.39 | 24.95 | 0.58 |
| CFC1B | cripto, FRL-1, cryptic family 1B | pancreas, prostate, stomach | 2.49 | 4.36 | 0.57 |
| SLC39A2 | solute carrier family 39 (zinc transporter), member 2 | prostate, esophagus, skin | 13.22 | 23.85 | 0.55 |
| RBFOX3 | RNA binding protein, fox-1 homolog (C. elegans) 3 | prostate, brain, cervix, testis, urinary bladder, esophagus | 15.39 | 28.45 | 0.54 |
| MUC3A | mucin 3A, cell surface associated | colon, prostate, small intestine, duodenum, gallbladder | 29.95 | 56.16 | 0.53 |
| BEST3 | bestrophin 3 | prostate brain, testis esophagus | 3.34 | 6.46 | 0.52 |
| CYP4F31P | cytochrome P450, family 4, subfamily F, polypeptide 31, pseudogene | prostate, testis | 1.21 | 2.45 | 0.49 |
| CACNG4 | calcium channel, voltage-dependent, gamma subunit 4 | lung, prostate, brain | 11.81 | 24.47 | 0.48 |
| RPRM | reprimo, TP53 dependent G2 arrest mediator candidate | prostate, brain, stomach, adrenal, cervix, ovary, salivary gland | 6.66 | 13.81 | 0.48 |
| AL162431.1 | Uncharacterized protein | prostate, duodenum | 1.85 | 3.89 | 0.48 |
| POTEI | POTE ankyrin domain family, member I | prostate, testis | 1.28 | 2.74 | 0.47 |
| CFC1 | cripto, FRL-1, cryptic family 1 | pancreas, prostate, stomach | 3.16 | 6.96 | 0.45 |
| ATP2A1 | ATPase, Ca++ transporting, cardiac muscle, fast twitch 1 | prostate, esophagus | 17.79 | 39.97 | 0.45 |
| SMTNL1 | smoothelin-like 1 | prostate, esophagus | 3.27 | 7.53 | 0.43 |
| RP11-664D7.4 | HCG1787533; Uncharacterized protein | pancreas, lung, prostate | 3.22 | 7.56 | 0.43 |
| SIM2 | single-minded homolog 2 (Drosophila) | kidney, prostate, stomach, esophagus | 8.32 | 20.04 | 0.42 |
| CNTNAP2 | contactin associated protein-like 2 | prostate, brain | 10.61 | 28.38 | 0.37 |
| SIX1 | SIX homeobox 1 | prostate, fat, salivary gland | 13.14 | 37.25 | 0.35 |
| MASP1 | mannan-binding lectin serine peptidase 1 (C4/C2 activating component of Ra-reactive factor) | liver, prostate, brain, cervix, heart | 31.60 | 89.88 | 0.35 |
| HOXD11 | homeobox D11 | colon, kidney, prostate, cervix | 4.95 | 14.19 | 0.35 |
| AMTN | amelotin | prostate, stomach | 2.14 | 6.16 | 0.35 |
| LRTM1 | leucine-rich repeats and transmembrane domains 1 | prostate, spleen, esophagus | 1.67 | 5.41 | 0.31 |
| SLN | sarcolipin | prostate, esophagus, heart | 52.78 | 172.10 | 0.31 |
| ELOVL2 | ELOVL fatty acid elongase 2 | liver, prostate, brain, placenta, testis | 7.61 | 28.53 | 0.27 |
| CD177 | CD177 molecule | colon, prostate, bonemarrow | 38.99 | 148.46 | 0.26 |
| NTNG2 | netrin G2 | prostate, brain, bonemarrow | 5.77 | 22.26 | 0.26 |
| ENO3 | enolase 3 (beta, muscle) | liver, prostate, esophagus, heart | 99.06 | 383.29 | 0.26 |
| CLGN | calmegin | prostate, testis, heart | 21.97 | 86.58 | 0.25 |
| SLITRK5 | SLIT and NTRK-like family, member 5 | prostate, brain, thyroid ,salivarygland | 2.47 | 9.80 | 0.25 |
| HOXD10 | homeobox D10 | colon, kidney, prostate, cervix | 9.50 | 39.92 | 0.24 |
| ACOXL | acyl-CoA oxidase-like | lung, prostate, testis, urinary bladder | 3.44 | 14.52 | 0.24 |
| TMEM178B | transmembrane protein 178B | prostate, brain, thyroid, heart | 2.72 | 11.92 | 0.23 |
| CLVS2 | clavesin 2 | prostate, brain | 2.22 | 9.88 | 0.22 |
| CRHR1 | corticotropin releasing hormone receptor 1 | prostate, brain, fat, cervix, skin | 1.14 | 5.10 | 0.22 |
| DNAH8 | dynein, axonemal, heavy chain 8 | prostate, testis | 1.67 | 7.66 | 0.22 |
| CDH10 | cadherin 10, type 2 (T2-cadherin) | prostate, brain | 4.79 | 21.95 | 0.22 |
| C1orf170 | chromosome 1 open reading frame 170 | prostate, esophagus, heart, skin | 4.61 | 21.92 | 0.21 |
| KCNJ3 | potassium inwardly-rectifying channel, subfamily J, member 3 | kidney, prostate, brain, small intestine, duodenum, heart | 3.65 | 17.50 | 0.21 |
| CAV3 | caveolin 3 | prostate, esophagus, heart | 3.43 | 16.80 | 0.20 |
