# Supplementary material for: Analysis of the Human Prostate-Specific Proteome Defined by Transcriptomics and Antibody-Based Profiling Identifies TMEM79 and ACOXL as Two Putative, Diagnostic Markers in Prostate Cancer
Source: PLoS One. 2015 Aug 3;10(8):e0133449. doi: 10.1371/journal.pone.0133449 (PMC4523174; doi:10.1371/journal.pone.0133449)
Supplement: S3 Table — (DOCX) [file pone.0133449.s005.docx]

**Supplementary Table 3: Enhanced genes in the Prostate defined as having a 5-fold higher FPKM level in prostate as compared to the average FPKM value of all the 27 tissues**

| **Gene name** | **Description** | **Category** | **Mean Prostate FPKM** | **Max FPKM in other tissue** | **Tissue-specific score** |
| --- | --- | --- | --- | --- | --- |
| TBL1Y | transducin (beta)-like 1, Y-linked | Enhanced | 11.73 | 0.35 | 4.64 |
| COL9A1 | collagen, type IX, alpha 1 | Enhanced | 10.41 | 4.16 | 4.60 |
| MYBPH | myosin binding protein H | Enhanced | 13.77 | 1.05 | 4.14 |
| SLC30A4 | solute carrier family 30 (zinc transporter), member 4 | Enhanced | 8.85 | 11.60 | 3.91 |
| MYOD1 | myogenic differentiation 1 | Enhanced | 17.11 | 0.32 | 3.85 |
| ANO7 | anoctamin 7 | Enhanced | 14.83 | 29.37 | 3.79 |
| RP11-1012A1.4 |  | Enhanced | 9.71 | 0.30 | 3.49 |
| ATP1B4 | ATPase, Na+/K+ transporting, beta 4 polypeptide | Enhanced | 16.59 | 0.65 | 3.48 |
| CPNE4 | copine IV | Enhanced | 14.72 | 15.62 | 3.38 |
| FOXA1 | forkhead box A1 | Enhanced | 11.69 | 33.88 | 3.29 |
| NLRP9 | NLR family, pyrin domain containing 9 | Enhanced | 9.25 | 0.46 | 3.15 |
| GLB1L3 | galactosidase, beta 1-like 3 | Enhanced | 11.20 | 5.23 | 2.87 |
| STEAP1 | six transmembrane epithelial antigen of the prostate 1 | Enhanced | 8.38 | 33.66 | 2.86 |
| CHRNA1 | cholinergic receptor, nicotinic, alpha 1 (muscle) | Enhanced | 8.96 | 0.98 | 2.80 |
| ALOX15B | arachidonate 15-lipoxygenase, type B | Enhanced | 13.37 | 22.17 | 2.76 |
| CDH26 | cadherin 26 | Enhanced | 9.70 | 5.85 | 2.67 |
| NWD1 | NACHT and WD repeat domain containing 1 | Enhanced | 10.20 | 2.68 | 2.58 |
| TULP1 | tubby like protein 1 | Enhanced | 6.87 | 0.44 | 2.52 |
| ADRA1D | adrenoceptor alpha 1D | Enhanced | 8.01 | 1.21 | 2.49 |
| C7orf63 | chromosome 7 open reading frame 63 | Enhanced | 5.15 | 7.63 | 2.42 |
| ALDH1A3 | aldehyde dehydrogenase 1 family, member A3 | Enhanced | 7.71 | 53.60 | 2.36 |
| CPLX3 | complexin 3 | Enhanced | 14.46 | 15.54 | 2.35 |
| SHROOM1 | shroom family member 1 | Enhanced | 5.39 | 14.59 | 2.29 |
| BMPR1B | bone morphogenetic protein receptor, type IB | Enhanced | 9.11 | 11.61 | 2.28 |
| P2RX2 | purinergic receptor P2X, ligand-gated ion channel, 2 | Enhanced | 6.70 | 2.60 | 2.25 |
| DUPD1 | dual specificity phosphatase and pro isomerase domain containing 1 | Enhanced | 14.03 | 0.72 | 2.23 |
| PPP1R27 | protein phosphatase 1, regulatory subunit 27 | Enhanced | 12.03 | 2.43 | 2.23 |
| TMPRSS2 | transmembrane protease, serine 2 | Enhanced | 7.70 | 140.60 | 2.17 |
| DDIT4L | DNA-damage-inducible transcript 4-like | Enhanced | 6.75 | 8.89 | 2.16 |
| SLC2A12 | solute carrier family 2 (facilitated glucose transporter), member 12 | Enhanced | 5.49 | 11.90 | 2.11 |
| ABCC4 | ATP-binding cassette, sub-family C (CFTR/MRP), member 4 | Enhanced | 6.94 | 23.03 | 2.11 |
| TMSB15A | thymosin beta 15a | Enhanced | 5.99 | 5.92 | 2.10 |
| SPDEF | SAM pointed domain containing ets transcription factor | Enhanced | 11.98 | 52.38 | 2.08 |
| NEB | nebulin | Enhanced | 8.24 | 18.01 | 2.07 |
| CRISPLD1 | cysteine-rich secretory protein LCCL domain containing 1 | Enhanced | 5.65 | 14.99 | 2.05 |
| LRRC24 | leucine rich repeat containing 24 | Enhanced | 5.25 | 1.26 | 2.04 |
| NPPC | natriuretic peptide C | Enhanced | 6.79 | 2.12 | 1.94 |
| AGBL1 | ATP/GTP binding protein-like 1 | Enhanced | 11.82 | 0.93 | 1.93 |
| SLC15A2 | solute carrier family 15 (H+/peptide transporter), member 2 | Enhanced | 6.38 | 21.80 | 1.91 |
| RAB3B | RAB3B, member RAS oncogene family | Enhanced | 7.64 | 4.91 | 1.90 |
| PMEPA1 | prostate transmembrane protein, androgen induced 1 | Enhanced | 6.05 | 52.22 | 1.73 |
| CWH43 | cell wall biogenesis 43 C-terminal homolog (S. cerevisiae) | Enhanced | 8.73 | 24.73 | 1.69 |
| CA3 | carbonic anhydrase III, muscle specific | Enhanced | 10.02 | 63.67 | 1.69 |
| SLC35F4 | solute carrier family 35, member F4 | Enhanced | 8.59 | 1.82 | 1.67 |
| MESP1 | mesoderm posterior 1 homolog (mouse) | Enhanced | 9.98 | 13.46 | 1.66 |
| ARHGEF38 | Rho guanine nucleotide exchange factor (GEF) 38 | Enhanced | 5.48 | 4.44 | 1.65 |
| PRDM8 | PR domain containing 8 | Enhanced | 5.97 | 13.51 | 1.58 |
| RP11-1102P16.1 | Uncharacterized protein | Enhanced | 9.62 | 0.96 | 1.55 |
| AC106722.1 | HCG1806964, isoform CRA_a; Uncharacterized protein; cDNA FLJ55426; cDNA, FLJ79414 | Enhanced | 7.04 | 2.58 | 1.52 |
| C8orf22 | chromosome 8 open reading frame 22 | Enhanced | 14.07 | 5.24 | 1.51 |
| AC005537.2 |  | Enhanced | 6.19 | 0.97 | 1.50 |
| C9orf92 | chromosome 9 open reading frame 92 | Enhanced | 11.00 | 0.79 | 1.49 |
| CPA6 | carboxypeptidase A6 | Enhanced | 9.07 | 2.85 | 1.47 |
| ALX4 | ALX homeobox 4 | Enhanced | 6.59 | 0.79 | 1.45 |
| GATA2 | GATA binding protein 2 | Enhanced | 5.17 | 36.66 | 1.44 |
| HIST1H2AE | histone cluster 1, H2ae | Enhanced | 5.64 | 10.28 | 1.43 |
| HIST2H2BF | histone cluster 2, H2bf | Enhanced | 5.28 | 8.18 | 1.40 |
| STAC3 | SH3 and cysteine rich domain 3 | Enhanced | 7.95 | 32.45 | 1.34 |
| STAC | SH3 and cysteine rich domain | Enhanced | 5.88 | 8.91 | 1.32 |
| PRKAG3 | protein kinase, AMP-activated, gamma 3 non-catalytic subunit | Enhanced | 9.93 | 1.30 | 1.29 |
| MYOZ3 | myozenin 3 | Enhanced | 5.84 | 6.86 | 1.29 |
| EVX1 | even-skipped homeobox 1 | Enhanced | 10.99 | 1.64 | 1.27 |
| SLC14A1 | solute carrier family 14 (urea transporter), member 1 (Kidd blood group) | Enhanced | 6.59 | 41.94 | 1.25 |
| LPAR3 | lysophosphatidic acid receptor 3 | Enhanced | 5.28 | 12.90 | 1.24 |
| OR51E1 | olfactory receptor, family 51, subfamily E, member 1 | Enhanced | 7.84 | 7.92 | 1.24 |
| PDE11A | phosphodiesterase 11A | Enhanced | 5.44 | 5.76 | 1.24 |
| ASTN2 | astrotactin 2 | Enhanced | 5.48 | 22.59 | 1.22 |
| GUCY2D | guanylate cyclase 2D, membrane (retina-specific) | Enhanced | 5.17 | 0.98 | 1.20 |
| RFPL1 | ret finger protein-like 1 | Enhanced | 8.00 | 0.90 | 1.17 |
| ARHGAP6 | Rho GTPase activating protein 6 | Enhanced | 5.12 | 31.76 | 1.15 |
| GABRG3 | gamma-aminobutyric acid (GABA) A receptor, gamma 3 | Enhanced | 9.31 | 7.21 | 1.15 |
| MKX | mohawk homeobox | Enhanced | 6.28 | 10.87 | 1.06 |
| IRX4 | iroquois homeobox 4 | Enhanced | 10.04 | 4.27 | 1.06 |
| GLIS1 | GLIS family zinc finger 1 | Enhanced | 5.40 | 2.58 | 1.05 |
| RPE65 | retinal pigment epithelium-specific protein 65kDa | Enhanced | 8.54 | 1.42 | 1.04 |
| ACTG2 | actin, gamma 2, smooth muscle, enteric | Enhanced | 5.38 | 1166.37 | 1.04 |
| ISL1 | ISL LIM homeobox 1 | Enhanced | 7.15 | 9.10 | 1.03 |
| EFCAB12 | EF-hand calcium binding domain 12 | Enhanced | 6.35 | 6.43 | 1.01 |
| SELE | selectin E | Enhanced | 5.65 | 25.53 | 1.00 |
| COL2A1 | collagen, type II, alpha 1 | Enhanced | 5.79 | 2.62 | 0.97 |
| RYR1 | ryanodine receptor 1 (skeletal) | Enhanced | 5.59 | 4.15 | 0.97 |
| EVX2 | even-skipped homeobox 2 | Enhanced | 9.44 | 1.65 | 0.93 |
| RP11-481A20.11 | Uncharacterized protein | Enhanced | 9.14 | 2.81 | 0.92 |
| SYT7 | synaptotagmin VII | Enhanced | 5.12 | 52.87 | 0.91 |
| OPRK1 | opioid receptor, kappa 1 | Enhanced | 9.15 | 2.37 | 0.91 |
| ACSM1 | acyl-CoA synthetase medium-chain family member 1 | Enhanced | 5.62 | 6.45 | 0.91 |
| HIST1H2BF | histone cluster 1, H2bf | Enhanced | 6.27 | 3.03 | 0.88 |
| PCP4 | Purkinje cell protein 4 | Enhanced | 6.18 | 267.33 | 0.87 |
| CPAMD8 | C3 and PZP-like, alpha-2-macroglobulin domain containing 8 | Enhanced | 5.56 | 12.33 | 0.75 |
| MYOZ1 | myozenin 1 | Enhanced | 7.98 | 48.97 | 0.73 |
| MYADML2 | myeloid-associated differentiation marker-like 2 | Enhanced | 6.06 | 1.96 | 0.70 |
| PABPC1L2A | poly(A) binding protein, cytoplasmic 1-like 2A | Enhanced | 7.33 | 4.43 | 0.61 |
| SLC7A3 | solute carrier family 7 (cationic amino acid transporter, y+ system), member 3 | Enhanced | 5.90 | 3.90 | 0.60 |
| SALL3 | sal-like 3 (Drosophila) | Enhanced | 6.38 | 2.97 | 0.57 |
| MYLK2 | myosin light chain kinase 2 | Enhanced | 5.02 | 5.45 | 0.55 |
| DET1 | de-etiolated homolog 1 (Arabidopsis) | Enhanced |  | 0.00 |  |
